# Integration of cognitive behavioral therapy and mobile health applications among university students with depression: a qualitative study

**DOI:** 10.1080/17482631.2026.2697390

**Published:** 2026-07-15

**Authors:** Li-Ting Chen, Hsin-Tien Hsu, Meei-Fang Lou, Chia-Yi Wu, Cheng-Han Lin, Yueh-Hsiu Lin

**Affiliations:** a School of Nursing, College of Nursing, Kaohsiung Medical University, Kaohsiung, Taiwan, ROC; b School of Nursing, College of Medicine, National Taiwan University, Taipei, Taiwan, ROC; c Department of Health-Business Administration, School of Nursing, Fooyin University, Kaohsiung, Taiwan, ROC; d Department of Medical Research, Kaohsiung Medical University Hospital, Kaohsiung, Taiwan

**Keywords:** Cognitive behavioral therapy, depression, help-seeking, mobile applications, telemedicine

## Abstract

**Background:**

Most applications for depression lack comprehensive theoretical integration and qualitative assessments of university students’ needs remain insufficient.

**Objective:**

This study aimed to explore the needs and experiences of university students with depressive symptoms and develop a theory-driven app design framework tailored to the target population.

**Methods:**

A post-positivist qualitative framework was used to recognize the value of subjective experience. Semi-structured interviews were conducted with 32 students with moderate to moderately severe depression. Reflexive thematic analysis was used to identify themes in the data.

**Results:**

Three themes emerged: app design, help-seeking processes, and core features of cognitive behavioral therapy. Students emphasized the importance of discreet, user-friendly design, such as positive naming, privacy protection, and flexible reminder functions. Although some expressed concerns regarding the empathy and reliability of artificial intelligence, others valued its anonymity and capacity to provide immediate support. Regarding theoretical integration, participants considered monitoring emotions and physical sensations essential but also highlighted the need for diverse and personalized methods. The conceptualization of self-monitoring data was considered useful for facilitating clinical consultations.

**Conclusion:**

Students considered theory-based health education as effective for improving mental health knowledge and promoting help-seeking awareness. The findings support clinical decision-making in developing more effective digital tools.

## 1. Introduction

Based on cognitive behavioural therapy (CBT), the modification of maladaptive cognitions is hypothesised to improve emotional regulation and behaviour, thereby promoting psychological well-being (Dobson & Dozois, 2019). Psychological distress often emerges from interpretations of events influenced by prior experiences and ingrained schemas (American Psychological Association, 2017). Although events cannot be changed, their appraisal can, thereby supporting behavioural and emotional improvements through self-regulation. Notably, CBT, one of the most widely applied psychological models, posits that cognition influences behaviour and can be modified, and that behavioural change is based on restructuring (Beck, 2011). Distorted thoughts contribute to emotional disorders; thus, altering them can reduce symptoms. Contrary to pharmacological interventions, CBT addresses the cognitive basis of symptoms.

CBT interventions have proven effective (Dobson & Dozois, 2019). Nevertheless, depressive disorders remain prevalent; in 2021, depressive disorders were the second leading cause of years lived with disability (YLDs) among global people across all ages in 2021, with more than 332 million people globally (6.2% of total cause YLDs). While the demographics were distributed across the entire lifespan, depressive disorders were most common among females between the age groups of 15–19 years (GBD, 2024). Longitudinal research indicated a four-year period prevalence of 36.7% for 12-month probable major depressive episodes among university students (Nunnink et al., 2025). These students faced stressful life events, including high risk factors such as recent breakups/arguments, academic challenges, financial difficulties, and social stress, while also showing associations with Adverse Childhood Experiences (Barbayannis et al., 2022; Nunnink et al., 2025).

To address these issues among university students, mobile health (mHealth) applications have seen increasing use. These applications help improve self-management of depression, providing monitoring and care guidance (GBD, 2024). *Anonymous* (Nunnink et al., 2025) found significant symptom reduction after four weeks of using such an app, showing improvements across mild to moderate-to-severe cases, with greater effects among those with mild symptoms. These findings are consistent with those of Simblett et al. (2019), Barbayannis et al. (2022), who observed that, although students initially accepted app-based tools, engagement declined as symptoms worsened. Theoretical foundation is essential to clarify the mechanisms linking app use and symptom change. A CBT-based framework supports the development of components, evaluation criteria, and adaptive care (Nunnink et al., 2025).

However, most depression apps lack comprehensive theoretical integration, leading them to yield inconsistent outcomes. Many use commercial platforms that partially reflect CBT principles (Bantjes et al., 2024; Liu et al., 2022; Sabour et al., 2023). Although some show short-term improvement, the effects often diminish by weeks eight and twelve (Deady et al., 2023; Raevuori et al., 2021). Interactive features frequently lack personalisation or relevance to symptom severity (Bernstein et al., 2025). Furthermore, help-seeking functions typically list healthcare institutions but do not provide real-time guidance (Silfee et al., 2021). Although mood diary functions are consistent with emotional monitoring, they show limited impact on self-awareness, possibly due to the isolated use of CBT aspects (Danieli et al., 2022; Sabour et al., 2023).

In response, this study developed a CBT-based app consistent with theoretical constructs and customised to university students. It evaluated the app’s mid- to long-term effects on depressive symptoms, help-seeking attitudes, and emotional self-awareness. Furthermore, it examined how theoretical mechanisms function within the app to inform a model of precision care integrated with campus resources.

## 2. Background

### 2.1. Cognitive behavioural therapy theory and the outlook of mHealth applications

CBT is a well-established psychological framework that emphasises the interrelationship among cognition, emotion, and behaviour (Beck, 2011; Dobson & Dozois, 2019). CBT is an action-oriented and problem-centred framework. Its core theoretical mechanism involves guiding patients to identify their irrational beliefs, thereby facilitating cognitive restructuring. Furthermore, through specific goal setting, this approach enables patients to integrate their altered beliefs into their behaviours (Dobson & Dozois, 2019). Huguet et al. (2016) proposed CBT’s 10 concepts for depression: (1) education about depression, (2) explanation of the model, (3) depression rating, (4) monitoring cognition, (5) monitoring emotions, (6) monitoring physical sensations, (7) monitoring behaviours, (8) conceptualisation, (9) behavioural techniques, and (10) cognitive techniques. Its core premise is that modifying maladaptive cognitions improves emotional regulation and behavioural responses. Digital CBT-based interventions, particularly mHealth apps, have gained popularity; however, most incorporate a limited set of CBT principles. For example, Huguet et al. (2016) found that 15% of mental health apps were theory-driven, and many were not consistent with comprehensive CBT. Martinengo et al. (2021) reported that, although 25.5% of evaluated apps included four of CBT’s 10 main concepts, their theoretical mechanisms were rarely articulated. *Anonymous* (Nunnink et al., 2025) further noted that, among apps for university students with depression, 57.1% adopted CBT frameworks; however, few clarified how the concepts were operationalized. The investigator’s prior doctoral research also highlighted these gaps, demonstrating insufficient explanatory power due to the absence of a complete CBT structure. These findings underscore the need for theory-integrated digital interventions that are based on the comprehensive scope of CBT.

### 2.2. mHealth needs among university students with depression

mHealth technologies have attracted attention as scalable approaches for preventing and managing depression among university students (Madrid-Cagigal et al., 2025). Delivered through smartphone apps, text tools, and web platforms, they provide flexible and interactive support customised to young adults (Leong et al., 2022; Madrid-Cagigal et al., 2025). Only 20–40% of students access traditional services due to stigma, limited access, or scheduling constraints (Fitzsimmons-Craft et al., 2021). mHealth provides low-threshold alternatives whereas randomised controlled trials have reported improvements in depressive severity, well-being, and social connectedness (Jayaraj et al., 2025).

Effective engagement depends on user-centred design features such as usability, intuitiveness, personalisation, privacy safeguards, and dynamic feedback (Hoglund et al., 2025). Features that incorporate personalised recommendations, reminders, and customised interactions, particularly those that use artificial intelligence or machine learning, support better emotional monitoring and real-time responsiveness (McCarthy & Yan, 2024). Structured tools such as goal setting, mood journaling, and guided CBT-based modules further enhance self-regulation and reduce symptoms (Bendtsen et al., 2020). The asynchronous and private nature of mHealth increases appeal, especially among students concerned about stigma or time restrictions (Hoglund et al., 2025). However, issues persist regarding privacy, data security, and the need for hybrid models that combine digital and human support, such as peer engagement or professional counselling, when it comes to enhancing adherence and therapeutic relationship (Salamanca-Sanabria et al., 2020).

### 2.3. University students’ perspectives on app design

Students prioritise specific interface and aesthetic features when engaging with mental health apps. Elements such as colour palette, layout, visual aesthetics, sustainability, and engaging content strongly influence appeal (Tamtama et al., 2020). Interactive functions also sustain engagement. Preferred features include quizzes, flip-card modules, educational videos, and personalised feedback. For example, apps may assess psychological symptoms using scales and provide algorithmically generated care recommendations (Rainisch et al., 2022).

Emotional resonance is equally essential. Empathic design enables visual and emotional engagement, thereby strengthening relationships between users, services, and personal experience. Immersive multimedia, such as narrative visuals and auditory storytelling, can increase affective engagement (AnNing et al., 2024). Gamification further enhances motivation through features such as entertainment value, structured gameplay, goal-setting, feedback, mission unlocking, achievement perception, challenges, scenario-based problem-solving, social connectivity, and narrative storytelling (Subhash & Cudney, 2018). Users’ perceptions of app quality also depend on the credibility of the development team. Involvement of professionals, alignment of functions with end-user needs, and ease of navigation are critical for usability, acceptability, and effectiveness in managing depressive symptoms (Narrillos-Moraza et al., 2022).

### 2.4. Literature gap

There is a lack of mobile interventions tailored to precision mental health care for university students. Precision health aims to deliver personalised care based on biological, behavioural, and sociocultural factors, focusing on symptom monitoring, early risk identification, and self-management (Shun, 2023). This study leveraged CBT principles to propose a novel mobile app-based intervention model for supporting symptom tracking and behavioural change in an engaging and accessible format.

However, many depression-related apps lack condition-specific content (Shen, 2015) and theoretical models (Huguet et al., 2016; Martinengo et al., 2021). *Anonymous* (Nunnink et al., 2025) identified a major gap: although CBT-guided apps show short-term efficacy, none have comprehensively incorporated the full spectrum of CBT concepts. Therefore, this study aimed to design an app guided by these concepts, incorporating aspects that have already been shown to be effective while integrating those that have not been tested. The design also incorporates students’ preferences for app functions, interfaces, and usability, thereby enhancing willingness to engage and conveying CBT strategies in an accessible manner.

## 3. Methods

### 3.1. Aim

This study aimed to explore the application of CBT theory in mobile mental health interventions through a qualitative research approach. The objectives were to gain a comprehensive understanding of the needs and experiences of university students with depressive symptoms and develop a theory-driven app design framework customised to the target population.

### 3.2. Research design

This study was designed within a post-positivist qualitative framework, recognising the value of subjective experiences and diverse perspectives in knowledge construction. Post-positivism acknowledges that objective reality is not fully accessible and is mediated by the lens of individual interpretations and cultural contexts (Maksimović & Evtimov, 2023). Thus, a post-positivist framework is appropriate for discovering how technology facilitates interactions between individuals and CBT theory. With the incorporation of psychoeducation and self-care guidance to help users practice positive beliefs, behavioural changes required for autonomous mental health management can be achieved.

Three researchers conducted interviews with students to explore their experiences with mobile mental health tools. None of the authors had conflicts of interest related to the participants or study. Data collection was conducted between September 2024 and January 2025.

#### 3.2.1. Setting

The study was conducted in three universities of a similar nature in Taiwan. Participants were screened and enroled by the first author. The prospective design included students with depression, irrespective of prior app use. In-person interviews were conducted in school discussion rooms, each lasting up to 60 minutes. A semi-structured guide was used, and all sessions were audio-recorded. Participants were informed of their right to pause or terminate the interview at any point.

#### 3.2.2. Participants

The relevant inclusion criteria were self-reported depressive symptoms and a score of >10 on the Patient Health Questionnaire-9 (PHQ-9) (Additional details are provided in Appendix 1). Students had to be enroled in the participating universities. The relevant exclusion criterion was full-time employment. Those who provided written informed consent were included. Recruitment was voluntary and they were guaranteed that participation would not affect academic performance, relationships with advisors, or access to counselling services. Confidentiality was emphasised to minimise potential demand, discomfort, or harm.

### 3.3. Data collection

A post-positivist approach values subjective experience alongside qualitative insights to explain why certain quantitative trends occur. Thus, we used semi-structured interviews for a comprehensive exploration of participants’ experiences while focusing on how change happens (condition-specific content, mechanisms…) within the study. The interview framework comprised the following thematic subjects: (1) experiences with campus counselling; (2) barriers and facilitators to accessing resources; (3) use of mental health apps; (4) needs, functions, and interactions in app-based self-care; (5) factors influencing app selection; (6) help-seeking experiences; and (7) exploration of CBT’s 10 main concepts within an app (Additional details are provided in Appendix 1). Furthermore, prior to the formal data collection, the interview guide was indeed piloted with two participants to ensure the appropriateness, relevance, and clarity of the questions. Based on the pilot phase, participants suggested providing generation-appropriate examples for the 10 CBT concepts. Therefore, we incorporated tailored examples to better align with the participants' age, cultural background, and common vocabulary. These two pilot participants were also included in the final data analysis. Although guided by predetermined topics, the interviewer adapted questions based on participant responses (Dearnley, 2005). Interviews continued until data saturation was reached; this was defined as the point at which no new themes emerged, which was monitored throughout the data collection process. Although saturation typically occurred between 10 and 30 interviews (Guest et al., 2006), a two-phase interview approach was used for initial exploration and subsequent theme validation, reflecting the complexity of depressive symptoms.

### 3.4. Data analysis

Post-positivists use rigorous procedures to approximate the truth and minimise bias, which focuses on content analysis and inter-coder agreement. Thus, reflexive thematic analysis was adopted instead of content analysis to emphasise researcher subjectivity in interpreting, common experience, and developing meaning (Braun & Clarke, 2022, 2023). Two authors independently transcribed and coded the data, with oversight by YHL and FYL to ensure rigour. Analysis was conducted according to Braun and Clarke’s six-stage process. Transcriptions were imported into NVivo for coding, and data were compared and merged into thematic categories and concepts. An example of the code-to-theme development is presented in Table I.

**Table I.** Summary of interpretative theme, exemplary quotes, and implication for app design and care pathways.

Theme (name)	Theme definition	Key subthemes	Exemplar quotes
**Theme 1. “Discreet, controllable, and reassuring”: privacy and stigma management shape whether students will keep using the app**	Students’ sustained engagement depends on whether the app helps them manage disclosure risk and stigma through discretion, user control, and conditional trust.	**1.1 Blending in and staying in control**: packaging, naming, reminders as stigma-management strategies.	**C31** (optional lock; privacy boundary).
			**C10** (reminders can backfire; need to switch off).
			**C6** (scepticism about AI for severe issues).
		**1.2 Conditional trust in AI**: balancing privacy, safety, and a “human” feel.	**C11** (AI should listen + analyses + suggest).
		**1.3 “Helpful, but not exposing”**: novel features that support engagement while protecting privacy.	**C30** (preference-based AI interaction: listen vs advise).
**Theme 2. CBT must be low-burden and actionable in low mood: from “too hard to do” to guided micro-interventions**	CBT features are valued when they reduce cognitive/emotional load and remain doable during low mood through guided, stepwise micro-interventions.	**2.1 Low-friction monitoring without worsening distress** (quick, flexible recording).	**C1** (tags/text input; breathing support via prompts/music).
		**2.2 “Doable first steps”**: behavioural micro-interventions and selective tracking.	**C3** (techniques can worsen mood when not doable).
			**C9** (sleep tracking useful for doctor; acute distress needs immediate support).
		**2.3 CBT education as scaffolding**: concise, practical, visually supported.	**C13** (need visuals; pacing).
		**2.4 Conceptualisation as a bridge to care**: usable summaries for consultations.	**C7** (records aid recall; support judgement).
**Theme 3. Apps as a bridge—not a replacement: filling gaps created by wait times, stigma, and inadequate triage**	Apps are positioned as an “in-between” resource that provides timely, private support when services are delayed, disclosure is difficult, and triage is insufficient for urgent-but-non-life-threatening distress.	**3.1 “Getting through it” alone**: self-management as endurance.	**C3** (reluctance to ask for help; coping by staying busy).
		**3.2 “Access is fragile”**: mixed counselling experiences; high cost of persistence.	**C2** (drop-out when appointments unavailable; mismatch).
		**3.3 Disclosure under pressure**: competing demands, shame, stigma.	**C5** (administrative/emotional cost; family guilt).
		**3.4 The “in-between” gap**: inadequate triage outside clinic hours.	**C9** (need immediate support; ER non-response unless life-threatening; sleep info for doctor).

### 3.5. Integrity and motivation of the interview setting

Trustworthiness was assessed using Lincoln and Guba’s (1985) four criteria: credibility, transferability, dependability, and confirmability. Credibility was enhanced through interviewer training and joint initial sessions, with the corresponding author reviewing transcripts and processes. Regular meetings enabled peer debriefing. Transferability was supported by open-ended questioning and participant transcript checking. Dependability was ensured through explicit procedures and expert review of the analysis. Finally, confirmability was maintained by emphasising neutrality, practicing non-judgmental listening, and avoiding bias.

Recruitment challenges were addressed by expanding the research activities to online interviews and additional schools due to low participation. Participants were offered refreshments, interview fees, transport reimbursements, and transcript review honoraria to encourage engagement. These measures aimed to create a comfortable environment and sustain participation.

### 3.6. Ethical considerations

All participants were provided with detailed written informed consent after receiving full explanation of the research. The consent included details about the study purpose, procedures, potential risks and benefits, data protection measures, and participants' right to withdraw at any time. The study was approved by *[blinded for review]* (No. *[blinded for review]*, date of approval: *[blinded for review]*).

## 4. Results

**Figure 1.**
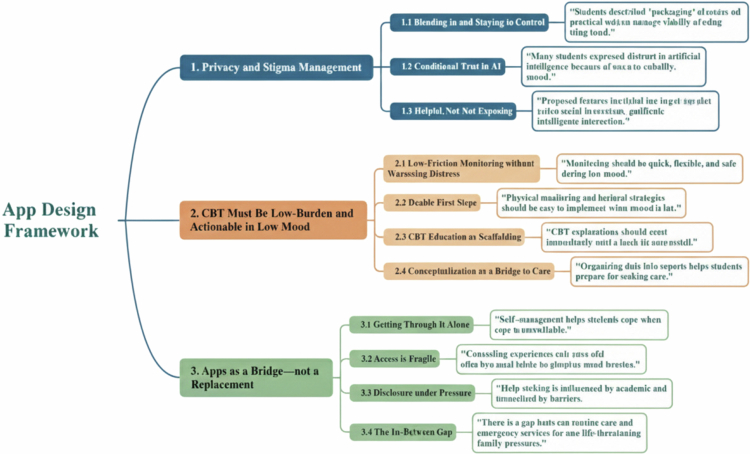
Qualitative analysis of user preferences for mental health applications.

A total of 32 students were interviewed, aged 19–32 years (mean: 21.1 years). Six (18.8%) and 26 (81.3%) were men and women, respectively; each completed one or two interviews. All participants completed the Patient Health Questionnaire-9; the average score was 14.6, indicating moderate to moderately severe depression (Table II). Thematic analysis identified the following main themes: “Discreet, controllable, and reassuring”, “CBT must be low-burden and actionable in low mood”, and “Apps as a bridge—not a replacement” (Figure 1).

**Table II.** Sample characteristics.

Characteristic		
Gender (female), *n* (%)	26	(81.3)
Age at consent (years), mean (SD)	21.2	(2.6)
PHQ-9 scale, mean (SD)	14.6	(4.4)
**PHQ-9 scale level, *n* (%)**		
Moderate depression	19	(59.4)
Moderately severe depression	7	(21.9)
Severe depression	6	(18.7)

PHQ-9: Patient Health Questionnaire-9.

### 4.1. “Discreet, controllable, and reassuring”: privacy and stigma management

Regarding the design of the app, students emphasised discreet presentation, privacy, and flexible reminders. Names and icons were optimistic and low-profile, and a password lock was widely supported. Reminders were helpful when customisable, although excessive notifications could deter use. Students’ views on AI were mixed: their concerns were based on empathy, accuracy, and privacy, yet many acknowledged AI’s value in providing some form of companionship, basic analysis, and suggestions. Desired functions included selective AI characters, adaptive communication modes, and proactive care.

#### 4.1.1. Packaging, naming, and reminders as stigma-management strategies

Students described “packaging” choices as practical ways to manage visibility and reduce the risk of being labelled, rather than as superficial design preferences. They preferred simple layouts, precise categorisation, and multiple input methods (including voice). Furthermore, it was indicated that the name and appearance of the app should not signal a mental health function; disease terms (e.g. “depression”) were discouraged. A low-profile icon and password lock were recommended. As one participant explained:

*C31: Individuals who know your phone password, such as family or a partner, can open the app. However, if you do not want others to know about the app, you may need to lock it. Users should be able to turn the lock on or off.*

Most students (*n* = 8) endorsed reminders but differed in their frequency of use. Some users supported daily prompts, while others preferred notifications after periods of non-use (e.g. five days). A minority preferred automated timing to match emotional fluctuations better. Importantly, students noted that reminders could backfire when they were feeling low, reinforcing the need for user control:

*C10: I would say ‘yes and no’ for reminders. They help with regular writing, but if I open the app when I feel down, a notification might worsen my mood. Users should be able to switch it off.*

#### 4.1.2. Conditional trust in AI: Balancing privacy, safety, and a “human” feel

Many students expressed distrust in artificial intelligence because of severe issues, citing privacy risks and a lack of empathy, although some indicated that they would use it for simpler tasks.

*C6: Most individuals prefer talking to a human. I am sceptical of artificial intelligence; I may use it for simple matters, but I am unsure if it can effectively address severe issues.*

Simultaneously, ten students valued artificial intelligence for companionship and support, while five highlighted its value regarding attentive listening and help in organising emotions. The anonymity provided by artificial intelligence was also noted to reduce self-consciousness. Finally, some students wanted artificial intelligence to analyse problems, provide suggestions, and timely prompts.

*C11: I expect something like a counsellor who listens and responds. Rather than ‘I see’, it should analyse my situation and suggest what I could do.*

#### 4.1.3. Novel features that support engagement while protecting privacy

Proposed features included audio input, short-video social interaction, gamification, artificial intelligence interaction, affiliations to professionals, and integrated records. Students wanted artificial intelligence that adapts to preferences (e.g. selectable personas, adjustable styles) and provides practical guidance when experiencing negative emotions.

*C30: Some individuals want to be heard; others wish to advise. Ask users what they prefer—attentive listening or feedback—before the conversation.*

### 4.2. “CBT must be low-burden and actionable in low mood”: From “too hard to do” to guided micro-interventions

Students considered monitoring and education based on CBT essential. They wanted diverse ways to record states and explicit, non-technical information. Cognitive and behavioural techniques were valued but considered difficult to apply during severe low moods. Those receptive to medical care wanted integrated reports to support clinical consultations.

#### 4.2.1. Low-friction monitoring without worsening distress

Students did not reject CBT-oriented self-monitoring; instead, they emphasised that monitoring must remain quick, flexible, and emotionally safe—especially during low mood. Fifteen students endorsed tracking emotions and cognition, including intensity. Preferred methods included short questionnaires, spectrum scales, brief notes, diary entries, emojis/tags, and speech-to-text. Some warned that recording during negative moods could intensify distress; a few suggested brief, frequent reports (every two to three days).

*C1: Text is clearer than voice notes. Tags such as ‘agitated’ or ‘numb’ could help.*

*C3: When I feel deficient, being told to use techniques can make me feel worse because I cannot do them.*

#### 4.2.2. Behavioural micro-interventions and selective tracking

Participants often described physical monitoring and behavioural strategies as “doable first steps” when mood and motivation were low. Ten students supported the occasional tracking of appetite, sleep, weight, and related behaviours through phone sensors or wearables. Such records were considered objective and helpful during medical visits, although consistency was more challenging during periods of low mood; additionally, sleep tracking accuracy was a concern.

*C9: If I can track my sleep, I can tell my doctor that I wake often, do not fall asleep easily, have nightmares, or lack deep sleep.*

Behavioural techniques (e.g. brief exercise, guided breathing) were generally considered acceptable and helpful when the mood allowed. Interactive aspects and rewards could sustain practice; however, their effectiveness varied depending on the participants’ emotional states.

*C1: When I am nervous, prompts or gentle music help me follow breathing instructions.*

#### 4.2.3. CBT education as scaffolding

Students were receptive to CBT explanations and depression-related education when content was designed to be immediately useful rather than comprehensive. They emphasised the need for concise, practical information delivered in accessible formats, with visuals and stories to maintain engagement and reduce cognitive load. Participants also requested clear guidance on care approaches and relaxation skills, suggesting that education should support action, not only understanding.

*C13: Short videos move too fast; text alone may be skipped. Adding pictures would help.*

In this sense, psychoeducation was framed as “scaffolding”: it should simplify and support decision-making during low mood, rather than adding information that users may not have the capacity to process.

#### 4.2.4. Conceptualisation as a bridge to care: Turning lived experience into usable summaries

Conceptualisation, organising emotional, physical, behavioural, and cognitive data into a report was valued by students who intended to seek care. Depression-related memory issues can hinder recall; thus, summaries could inform diagnosis and treatment.

*C7: Without immediate documentation, I forget details when I see the doctor; a record supports a more accurate judgement.*

Conceptualisation therefore functioned as an applied, low-burden CBT component that supports guided help-seeking: it translates lived experience into a format that can inform assessment and treatment, especially when low mood makes self-report difficult.

### 4.3. “Apps as a bridge—not a replacement”: Filling gaps created by wait times, stigma, and inadequate triage

Students’ accounts suggested that help-seeking was shaped by a combination of limited-service accessibility and socially situated barriers to disclosure. They described varied coping strategies such as attention shifting, keeping busy, prayer, and insufficient support from close contacts, whereas some endured difficulties alone or shared emotions anonymously. Experiences with counselling were mixed; waiting times and limited availability were common barriers. Concerns regarding medication and career implications were also noted. Academic and family stressors affected help-seeking, and shame or fear of stigma reduced disclosure. Students perceived gaps in triage systems and suggested that timely mobile support could provide a personal psychological emergency option.

#### 4.3.1. Self-management as endurance rather than recovery

Students described self-management as a way of enduring distress when formal or interpersonal support felt inaccessible. Coping strategies included music, videos, sleep, exercise, shopping, prayer, social-media breaks, drawing, and mind-wandering. Some students avoided issues or sought to “numb” themselves by staying busy; a few posted private updates or used chatbots as a form of expression.

*C3: I seldom ask for help. Others may not be able to do much; thus, I try to cope and keep busy.*

Together, these strategies reflect coping as “making it through” rather than resolving underlying difficulties, helping explain why a private, readily available digital option may feel more acceptable than immediate face-to-face help-seeking.

#### 4.3.2. Mixed counselling experiences and concerns about perceived usefulness

Participants’ narratives suggested that even when counselling was viewed positively, continued engagement was vulnerable to structural barriers and concerns about suitability and safety. On-campus counselling was viewed positively by some (supportive counsellors, comfortable settings), but many cited long waits and limited slots; others reported privacy concerns and unhelpful advice. Student outpatient services were appreciated for their subsidies and thorough assessments; however, some distrusted medication or avoided affiliated hospitals due to fears of potential employment repercussions. Off-campus care was sometimes rejected owing to side effects, impersonal attitudes, or initial misdiagnosis.

*C2: If I cannot secure an appointment, I stop going; I sometimes find my supervisor’s views difficult to accept.*

*C9: Doctors tend to prescribe medicine; when distress is acute, I may need immediate support rather than a pill that takes time to work.*

These accounts indicate that “help-seeking” involves sustained effort and uncertainty; when access is delayed or experiences feel misaligned with needs, students may disengage—reinforcing the perceived value of apps as interim support rather than replacements for professional care.

#### 4.3.3. Competing demands, shame, and stigma shaping help-seeking

Students described help-seeking decisions as negotiated within the pressures of academic life and family responsibilities. Academic workload, examinations, internships, and family factors (debt, conflict, bereavement, trauma, and stigma about mental illness) were common stressors. Many students avoided confronting their emotions, isolated themselves, or were ashamed of their negative records, which reduced their willingness to seek help.

*C5: Accessing psychiatric services involved many forms; I felt guilty about the time and emotional cost to my family.*

Importantly, these barriers were not only logistical; they were relational and moral experiences (e.g. guilt, shame, fear of being judged), suggesting that digital self-care may be appealing partly because it allows pacing, privacy, and control when disclosure feels risky.

#### 4.3.4. Inadequate triage for urgent but non-life-threatening distress

Students identified a perceived gap between routine outpatient care and emergency services. They reported long outpatient wait times, fixed clinic hours, and insufficient emergency responsiveness for non-life-threatening crises. Late-night hotlines could be unattended.

*C9: If you go to the emergency room and say, ‘I feel terrible’, little happens unless it is life-threatening; there is no immediate psychological help.*

This perceived triage gap clarifies the “bridge” role assigned to apps: students wanted timely, real-time support that can de-escalate distress and provide a sense of immediate containment while formal services remain inaccessible.

## 5. Discussion

This study examined the application of CBT theory in mobile mental health interventions and developed a theory-based app design framework tailored to university students with depressive symptoms. The findings highlighted the interaction between digital tools and students’ personal challenges. The findings also provided insights into how app design, help-seeking processes, and CBT features influence engagement.

Theoretical foundation is crucial for understanding the mechanisms that link app use and symptom changes. Although some of the students’ real-life experiences resonate with findings in existing research, minimal qualitative exploration has been conducted on the experience of using CBT-based apps in the context of mental health services. The findings are resonant with the theoretical features of CBT theory used in clinical contexts to identify experiences of depressive symptoms and facilitate emotional improvements through self-regulation (Chan, 2025; Li & Tang, 2024); the findings support behavioural personalisation for help-seeking in app usage (Hornstein et al., 2023).

However, by hearing the stories of students with depression, as well as their opinions and experiences with CBT-based framework integration and app usage, we understood how these experiences are related. The findings showed that user perceptions of CBT-app features are influenced by both user interaction design and theoretical interface needs and integration, thereby emphasising the importance of user-centred design. A successful CBT-based mental health app may provide diverse and personalised monitoring tools, consider users’ emotional states to provide customised support, deliver understandable and relatable health education content, and most critically, conceptualise self-monitoring data and integrate it as a clinical reference. Our findings also indicate a key boundary condition for CBT delivery via apps: when mood is severely low, cognitively demanding techniques may feel unworkable or even self-critical, whereas low-friction monitoring and guided behavioural micro-interventions may be more actionable. This supports a stepped, scaffolded approach that prioritises “doable first steps” and immediate usability before more effortful cognitive work. In this sense, our framework extends CBT-oriented app models by emphasising not only which CBT components are included, but how they should be paced, simplified, and integrated to sustain engagement under real-world constraints. Finally, CBT also informed not only the app design but also our interpretation of the qualitative findings. Students’ emphasis on low-burden monitoring, psychoeducation, “doable first steps,” and report-based summaries that integrate physiological indicators and self-monitoring data can be understood as pragmatic adaptations of core CBT processes under real-world constraints (e.g. low mood, stigma, and limited-service accessibility). In this way, CBT helped explain not only what features were preferred, but why these features may support engagement and guided help-seeking.

The innovative development of artificial intelligence is expected to support students who require mental health support. This is consistent with studies that suggest artificial intelligence experiences serve as a human touchpoint, providing users with empathy through active conversation and listening (Juquelier et al., 2025; Rubin et al., 2024). However, the findings highlighted a vital concern regarding artificial intelligence security and privacy, which may call into question the reliability of metal problem-solving strategies, such as data poisoning, information risk, and ethical implications. Furthermore, many students expressed distrust of artificial intelligence-assisted applications primarily due to concerns that artificial intelligence may not be able to handle severe mental health issues and apprehensions that backend records may pose privacy risks. Additionally, students believed that artificial intelligence’s responses lack the emotional warmth and empathy of humans; it only provides standard or rational suggestions, which often fail to satisfy users’ needs for emotional support. In addition to being a form of companionship, some students expected artificial intelligence to actively show care, such as providing reminders, analysing emotional changes, and proposing specific suggestions. Overall, students’ expectations for artificial intelligence applications were not limited to receiving emotional support and understanding. They also desired to receive practical assistance in problem analysis and solutions.

Students often struggle to cope with their emotions and predicaments on their own. They are reluctant to bother their friends or family and suspicious of the help that others can provide, which prevents them from seeking help (McKenzie et al., 2018). Participants stated that they keep themselves busy to escape or manage their negative emotions. Moreover, students seeking temporary help often experience a profound sense of helplessness and considerable emotional distress. However, immediate mental support is lacking, and existing mental health hotlines frequently fail to meet expectations or provide tangible assistance. We interpret these patterns as socially and institutionally situated rather than “mere user preferences.” In university and family contexts, stigma and concerns about being identified as needing mental health support can make disclosure feel risky, which helps explain students’ reliance on private, self-managed coping. Accordingly, privacy-by-design and discreet, controllable app features are not superficial design choices but pragmatic responses to the social consequences of visibility and judgement. Notably, participants spoke relatively little about social support and networks. We interpret this absence as meaningful rather than merely an omission: reluctance to “bother” others and doubts about the usefulness of interpersonal support may narrow relational help-seeking and increase reliance on solitary coping. These barriers appear not only logistical but also relational and moral (e.g. guilt, shame, and fear of being judged), which can make disclosure feel risky. Simultaneously, we acknowledge a potential unintended consequence: if CBT-based apps are designed solely as private self-help tools, they may inadvertently reinforce isolation. To mitigate this risk without compromising privacy, app design and implementation may consider optional relational pathways (e.g. user-controlled sharing of structured summaries with a clinician or trusted person, guided prompts to prepare help-seeking conversations, and clear triage, connection routes to campus or community services when distress escalates).

Furthermore, the emergency department primarily focuses on acute physical conditions, often providing insufficient attention to mental health issues. This creates a noticeable gap in services for cases that are between routine outpatient care and urgent emergencies. This “in-between” triage gap is especially salient for urgent but non-life-threatening distress, when students need immediate psychological containment but routine services are inaccessible. Thus, it is essential to consider individual needs and effectively transfer any type of mental health service to provide customised care according to a person’s mental status, thereby supporting them without causing additional stress.

According to this study, students considered CBT-based health education effective for improving mental health knowledge and promoting help-seeking awareness. Such apps may provide accurate and structured guidance and therefore address the inconsistent policy promotion and unreliable online information gaps. The theory-driven app framework provides practical direction for developers and offers clinicians insights into how digital tools can complement care and optimise the clinical application of mobile interventions. Under these constraints, mobile tools may serve as an “in-between” option that offers timely support and de-escalation while formal services remain out of reach, functioning as a bridge rather than a replacement for professional care.

### 5.1. Limitations and future research directions

This study has some limitations. The sample lacked demographic and geographic diversity, which restricted generalisability. Future work should include a broader student population from various institutions, regions, and cultural contexts. The study also relied on self-reported accounts rather than observational data, which may limit accuracy. The use of complementary objective measures could enhance validity. Finally, contextual factors such as cultural norms and service policies influenced perceptions, highlighting the need for further cross-cultural investigations of CBT-based app use.

### 5.2. Implications of the study

Students highlighted the importance of continuous monitoring. They observed that emotions and events are difficult to recall accurately, which can distort accounts in counselling and complicate diagnosis. Regular app use for recording mood and experiences enables timely documentation and generates valuable data for consultations. A CBT-based framework can also support clinical decision-making, encourage proactive help-seeking, and enhance engagement with mental health care.

## Data Availability

The data that support the findings of this study are available from the corresponding author, Yueh-Hsiu LIN, upon reasonable request.

## Trial registration numbers

The study was approved by the Research Ethics Committee of the Kaohsiung Medical University Hospital (No. KMUHIRB-E(I)-20230271, date of approval: December 20, 2023).

## Appendix 1. Instruments

1. Demographic Information.

Demographic characteristics included age and sex.

2. Primary Outcome Measure: Depressive symptoms.

We utilised the Patient Health Questionnaire-9 (PHQ-9) to assess depressive symptoms (Kroenke et al., 2001). The scale evaluates the frequency of depressive symptoms based on the nine criteria for major depressive disorder. It consists of 9 items in a four-point Likert-type format and requires 2–5 minutes to complete. The total scores range from 0 (no depressive symptoms) to 27 (severe depressive symptoms). The scale demonstrated excellent reliability, with a Cronbach’s *α* of .89 and test–retest reliability of .84 (Kroenke et al., 2001).

3. Semi-Structured Interview Framework.

The interview guide was developed based on the following seven-domain framework:

3.1 Experiences with campus counselling and mental health support
  - Mental health talks and workshops.
  - Psychological counselling.
  - Mental health clinic visits.
  - Meetings with academic advisors.
3.2 Barriers to and facilitators of accessing mental health resources.
3.3 Use of mental health apps.
3.4 Needs, functions, and interactions in app-based self-care.
  - What are your views on including a reminder function in the app?
  - If AI-related features were included, what kind of support would you want AI to provide?
  - Would you want the app to connect you with services such as video consultations or clinic appointment booking?
  - Under what circumstances would you want the app to provide support, such as during a late-night crisis or during a stable period?
3.5 Factors influencing app selection.
3.6 Help-seeking experiences.
  - New coping behaviours.
  - Appointment systems.
3.7 Exploration of the 10 main cognitive behavioural therapy (CBT) concepts within an app.

**Table:**

Core component	Example
Psychoeducation about depressive symptoms	Providing health education information and self-care guidance related to depressive symptoms
Explanation of cognitive behavioural therapy	Explaining why CBT may be useful and how it can support emotional and behavioural change
Monitoring depressive symptoms	Using numerical ratings or visual tools to help users understand their current mood and identify possible depressive symptoms
Monitoring cognitive states	Helping users reflect on thoughts related to low self-confidence, low self-worth, or excessive self-blame
Monitoring emotional responses	Recording emotional experiences such as crying, low mood, or emotional distress
Monitoring bodily sensations and somatic symptoms	Tracking experiences such as insomnia, reduced appetite, or other physical changes
Monitoring behavioural patterns	Recording behaviours such as withdrawal, avoidance, or reduced interpersonal interaction
Conceptual guidance on CBT skills	Explaining how cognitive and behavioural strategies can be applied in daily life
Behavioural techniques	Could you share your experience, if any, of using cognitive techniques—for example, practicing mindfulness—to help reframe or alter your thought patterns?
Cognitive techniques	Could you share any experiences you've had using behavioural techniques—for example, practicing breathing exercises or keeping a journal to track your own behaviours?
